# Steerable DROP-IN radioguidance during minimal-invasive non-robotic cervical and endometrial sentinel lymph node surgery

**DOI:** 10.1007/s00259-023-06589-3

**Published:** 2024-01-18

**Authors:** Matthias N. van Oosterom, Berta Diaz-Feijóo, Maria Isabel Santisteban, Núria Sánchez-Izquierdo, Andrés Perissinotti, Ariel Glickman, Tiermes Marina, Aureli Torné, Fijs W. B. van Leeuwen, Sergi Vidal-Sicart

**Affiliations:** 1 Interventional Molecular Imaging Laboratory, Department of Radiology, Leiden University Medical Center, Leiden, The Netherlands; 2 Gynecology Oncology Unit, Institute Clínic of Gynecology, Obstetrics, and Neonatology, Hospital Clinic of Barcelona, Barcelona, Spain; 3 Fundació de Recerca Clínic Barcelona - Institut d’Investigacions Biomèdiques August Pi I Sunyer (FRCB-IDIBAPS), Barcelona, Spain; 4 Faculty of Medicine, University of Barcelona, Barcelona, Spain; 5 Nuclear Medicine Department, Hospital Clínic of Barcelona, Barcelona, Spain; 6 Nuclear Medicine Department, Hospital Universitario Puerta del Mar, Cádiz, Spain

**Keywords:** Radioguided surgery, Sentinel lymph node, Cervical cancer, Endometrial cancer, Steerable instruments, Laparoscopic surgery

## Abstract

**Purpose:**

The recently introduced tethered DROP-IN gamma probe has revolutionized the way robotic radioguided surgery is performed, fully exploiting the nature of steerable robotic instruments. Given this success, the current first-in-human study investigates if the DROP-IN can also provide benefit in combination with steerable non-robotic instruments during conventional laparoscopic surgery, showing equivalence or even benefit over a traditional rigid gamma probe.

**Methods:**

The evaluation was performed in ten patients during laparoscopic cervical (*n* = 4) and endometrial (*n* = 6) cancer sentinel lymph node (SLN) procedures. Surgical guidance was provided using the hybrid, or bi-modal, SLN tracer ICG-^99m^Tc-nanocolloid. SLN detection was compared between the traditional rigid laparoscopic gamma probe, the combination of a DROP-IN gamma probe and a steerable laparoscopic instrument (LaproFlex), and fluorescence imaging.

**Results:**

The gynecologists experienced an enlarged freedom of movement when using the DROP-IN + LaproFlex combination compared to the rigid laparoscopic probe, making it possible to better isolate the SLN signal from background signals. This did not translate into a change in the SLN find rate yet. In both cervical and endometrial cancer combined, the rigid probe and DROP-IN + LaproFlex combination provided an equivalent detection rate of 96%, while fluorescence provided 85%.

**Conclusion:**

We have successfully demonstrated the in-human use of steerable DROP-IN radioguidance during laparoscopic cervical and endometrial cancer SLN procedures, expanding the utility beyond robotic procedures. Indicating an improved surgical experience, these findings encourage further investigation and consideration on a path towards routine clinical practice and improved patient outcome.

**Trial registration:**

HCB/2021/0777 and NCT04492995; https://clinicaltrials.gov/study/NCT04492995

## Introduction

In the management of many different cancer types, information on lymph node (LN) involvement helps to determine the disease status (i.e., node-positive N1 vs. node-negative N0) and the selection of optimal treatment planning. Although non-invasive imaging is increasingly contributing in the diagnosis of macroscopic tumor spread (e.g., ^18^F-FDG PET in cervical cancer) [1], surgical LN dissection and subsequent histopathological examination still provides the most accurate method to identify the presence of LN micro-metastases [2, 3]. Acting on the premise that cancer metastases pass through a group of gatekeeper LNs (i.e., the first draining LNs from the primary tumor side called the sentinel LNs (SLNs)), the SLN procedure was introduced to help reduce surgical complications associated with extensive LN dissections [4], while optimally identifying tumor-related lymphatics [5]. This procedure focuses on retrieval and detailed pathological investigation of these SLNs, facilitating ultra-staging of the important LN samples and, at the same time, helping to reduce the workload at pathology [6, 7]. The SLN procedure has become routine in various forms of cancer (e.g., breast cancer [8], melanoma [9], penile cancer [10]) and is increasingly being extended to other fields as well (e.g., prostate cancer [11], cervical cancer [12], endometrial cancer [13], bladder cancer [14], gastric cancer [15]), implementations that allow for a distinguishment to be made between patients who have oncological benefit from (invasive) nodal treatment (e.g., extensive dissection, debulking, cytoreduction, or radiotherapy) and those who would not benefit.

**Fig. 1:**
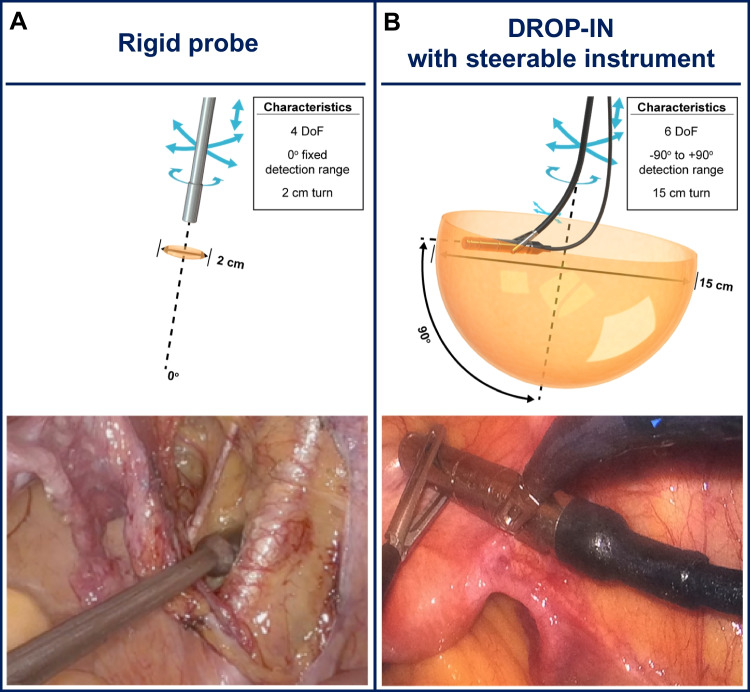
Overview of laparoscopic gamma probe applications investigated in this study. **A** Traditional rigid laparoscopic gamma probe, displaying available movement and intraoperative application. **B** DROP-IN probe usage with steerable laparoscopic instruments (i.e., DROP-IN + LaproFlex combination), displaying increased availability of movement and intraoperative application

To make it a patient-specific procedure, following the injection of a SLN-specific radiocolloid, preoperative lymphoscintigraphy and SPECT/CT imaging are used, imaging that helps to create a roadmap for surgical planning. Subsequent intraoperative localization of the identified targets is generally made possible using a gamma probe. This form of tracing delivers both in-depth audible and numerical feedback [16, 17]. By using hybrid, or bi-modal, SLN tracers (e.g., indocyanine green (ICG)-^99m^Tc-nanocolloid) [18], the radioguidance can be complemented with superficial visual fluorescence guidance. While initial SN work initially occurred during surgeries performed in an open surgery setting, technological advancements have supported implementation in a minimal-invasive laparoscopic surgery setting [19]. To allow for radioguidance in a laparoscopic setting, gamma probe manufacturers have had to adapt their probe designs, essentially elongating the gamma probe design and making it narrow enough to fit through, for example, 12–15 mm trocars [20]. Unfortunately, having the trocar as pivot point drastically limits the probe movement, thus reducing the degrees of freedom (DoF) from six for open surgery to four for laparoscopic surgery (see Fig. 1A). In robotic surgery, these challenges have been overcome following the introduction of tethered DROP-IN gamma probes [21]. These detectors are completely inserted in the abdomen through a trocar, where they can be picked up and manipulated with the (robotic) laparoscopic instruments [22]. Initial evaluations during robotic prostate cancer SLN procedures [23, 24] have indicated this improved maneuverability (6 DoF), which translated into an improved SLN detection rate compared to rigid laparoscopic gamma probes (100% vs 76%, respectively). The value of this DROP-IN probe concept was further underlined by the successful evaluation with different probe designs [21, 25–27] and in different robotic indications within urology (PSMA-targeted surgery (i.e., in primary [25, 28] and recurrent [29, 30] prostate cancer)) and gynecology (cervical cancer SLN surgery [31]).

The success of the DROP-IN probe in combination with the robotic steerable instruments makes one wonder if the technology can also provide benefit in combination with steerable (non-robotic) laparoscopic instruments. During laparoscopic cervical and endometrial cancer SLN procedures, we set out to evaluate if the combination of a DROP-IN gamma probe and a LaproFlex steerable laparoscopic instrument provides equivalence or even benefit over a traditional rigid gamma probe (see Fig. 1).

## Patients and methods

### Patient population and preoperative sentinel node mapping

This was a prospective pilot study. Ten patients with early-stage cervical cancer (*n* = 4) or low-to-intermediate-risk endometrial cancer (*n* = 6) were included between March and November 2022. All patients were scheduled for laparoscopic dissection of the primary tumor (i.e., radical hysterectomy) and SLN assessment. The inclusion criterion for cervical cancer was tumor stage IA1 with lymphovascular invasion, IA2 or IB1 (according to the International Federation of Gynecology and Obstetrics; FIGO 2018). Inclusion criteria for endometrial cancer were histology-proven endometrioid adenocarcinoma, but no involvement of the cervical stroma, and a myometrial invasion < 50% suspected. For both cancer types, the exclusion criteria were contraindication for surgical treatment, history of surgery or radiotherapy to nodal areas, and suspected metastatic disease based on preoperative imaging or preoperative biopsy.

Preoperative SLN mapping was based on both lymphoscintigraphy and SPECT/CT imaging using ICG-^99m^Tc-nanocolloid [18]. Hybrid tracer preparation and injection were performed as previously described for cervical [32] and endometrial [33] cancer. Due to logistic reasons, one patient received the radioactive-only tracer ^99m^Tc-nanocolloid. Eventual image reconstructions were examined by two nuclear medicine specialists and discussed with the surgical team prior to surgery. Patient characteristics and SLN mapping data are shown in Table 1. The study was approved by the local ethical committee (sub-study from NCT04492995—endometrial cancer; HCB/2021/0777—DROP-IN use in gynecological cancers) and informed consent was obtained from all individual patients included.

**Table 1:** Patient characteristics and intraoperative SLN detection rates

Variables	Values
Cervical cancer	
Patients included	4
Age (year), median (IQR)	39.5 (8.75)
FIGO 2018 tumor stage, *n* (%)	
- IA2	2 (50%)
- IB1	2 (50%)
Clinical node stage, *n* (%)	
- N0	4 (100%)
SLNs identified at preop imaging, suitable for surgical resection, *n* (median, IQR)	8 (2, 0.5)
Bilateral tracer drainage, *n* (% of total patients)	3 (75%)
SLNs found during surgery, *n* (median, IQR)	10 (2.5, 1.5)
SLNs detected with:	
- Rigid gamma probe, *n* (% of excised)	10 (100%)
- DROP-IN + LaproFlex combination, *n* (% of excised)	10 (100%)
- Fluorescence, *n* (% of excised)	7 (70%)
Metastases found at pathology, *n* (patient %)	0 (0%)
Endometrial cancer	
Patients included	6
Age (year), median (IQR)	60 (17.25)
FIGO tumor stage, *n* (%)	
- IA (grade G1)	1 (17%)
- IA (grade G2)	3 (50%)
- IA (grade G3)	2 (33%)
Clinical node stage, *n* (%)	
- N0	6 (100%)
SLNs identified at preop imaging, suitable for surgical resection, *n* (median, IQR)	14 (2.5, 1.75)
Bilateral tracer drainage, *n* (% of total patients)	4 (67%)
SLNs found during surgery, *n* (median, IQR)	13 (2.5, 1.75)
SLNs detected with:	
- Rigid gamma probe, *n* (% of pursued)	13 (93%)
- DROP-IN + LaproFlex combination, *n* (% of pursued)	13 (93%)
- Fluorescence, *n* (% of pursued)	10 (100%)*
Metastases found at pathology, *n* (% of patients)	1 (17%)

*Fluorescence was not used in one patient meaning only ten SNs were pursued that should be fluorescence-positive

### Surgical hardware and surgical procedure

Prior to the resection of the primary cancer (i.e., radical hysterectomy), a SLN dissection was performed. Preoperative SPECT/CT images served as a surgical roadmap on the number and location of the SLNs [34]. Both cervical and endometrial cancer procedures used a four trocar transperitoneal approach, with the patient in a Trendelenburg position. One 11-mm trocar was used for the laparoscopic camera, one 12-mm trocar for the gamma probes, and two 5-mm trocars for the laparoscopic instruments. After setting up the surgical field, the procedures started with the SLN localization. For intraoperative detection, a rigid traditional laparoscopic gamma probe (partially enclosed with sterile draping; Navigator, USSC, Norwalk, CT, USA) and a re-sterilizable DROP-IN gamma probe (see Fig. 1B; Drop-In Probe CXS-OP-DP, Crystal Photonics, Berlin, Germany) were used. As a steerable laparoscopic instrument, a 6 DoF short-fenestrated forceps LaproFlex instrument was used (DEAM, Amsterdam, The Netherlands). To support the comparison, individual SLN localizations were pursued with both the rigid gamma probe and the DROP-IN + LaproFlex combination. For confirmation of the SLN location, laparoscopic fluorescence imaging was used with either a Karl Storz fluorescence camera (KARL STORZ, Tuttlingen, Germany) or a Pinpoint fluorescence camera (Stryker, Kalamazoo, MI, USA). After excision of the SLN sample, confirmatory ex vivo measurements were performed with both gamma probe and fluorescence camera. The number and location of all SLNs excised were carefully reported, as well as their radioactive (counts) and fluorescent status (yes/no). Following surgery, all SLN specimens were carefully investigated for the presence of (micro-)metastases at histopathology, as described before [32, 33, 35].

The ten surgical procedures were performed by a total of four gynecologists, experienced in laparoscopic surgery. At the end of every procedure, the operating surgeon graded their experience (i.e., ease of use and available maneuverability) of the rigid gamma probe and DROP-IN + LaproFlex combination with one overall grade, ranging from 1 to 5 (where 5 was the most favorable grade).

## Results

### Preoperative imaging

**Fig. 2:**
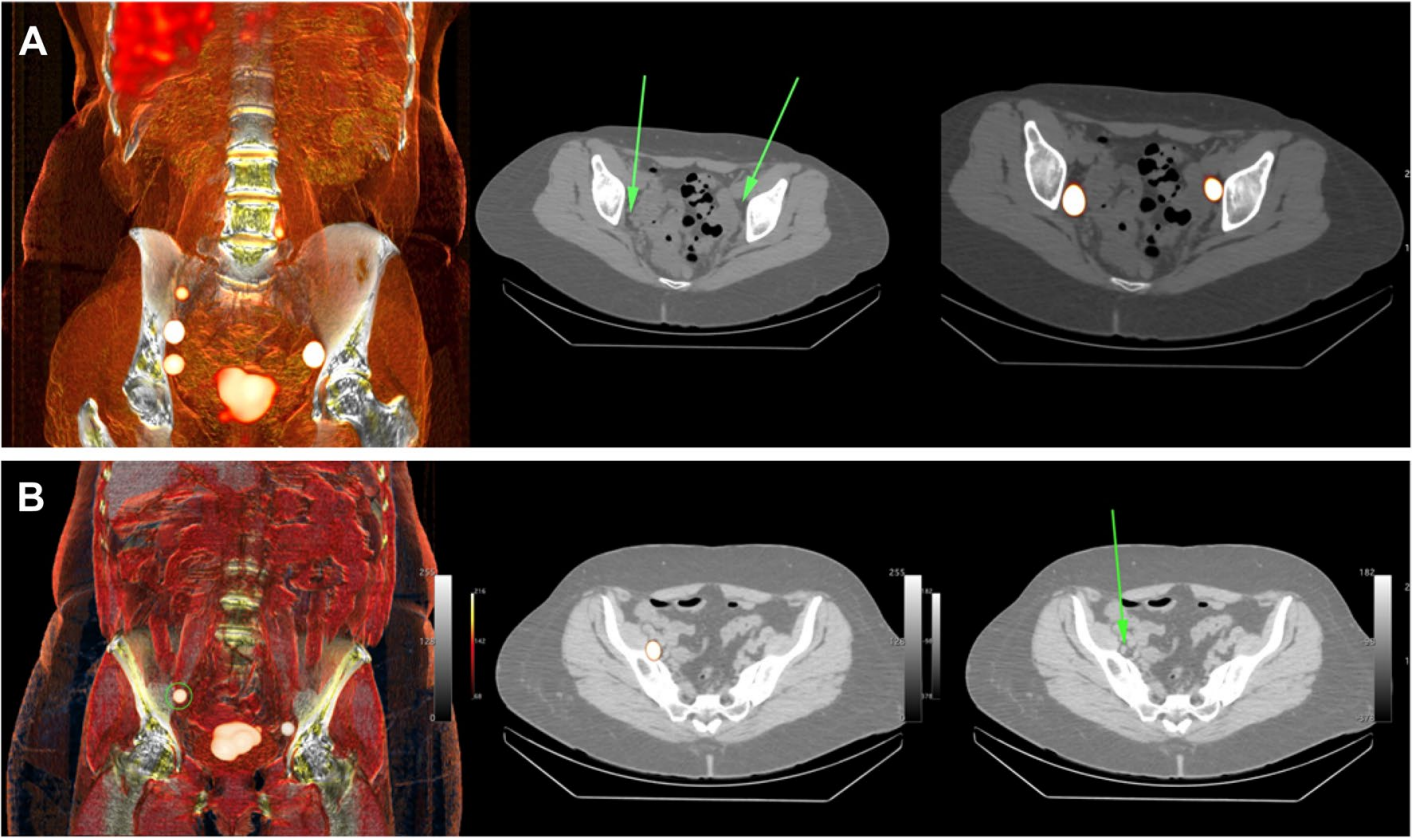
Preoperative sentinel lymph node mapping using nuclear imaging. **A** SPECT/CT imaging in cervix cancer, showing the sentinel node locations on a surface render overview (left) and detailed axial slices (middle and right). **B** SPECT/CT imaging in endometrial cancer, showing the sentinel node locations on a surface render overview (left) and detailed axial slices (middle and right)

Patient characteristics are summarized in Table 1. Median injected activity of ICG-^99m^Tc-nanocolloid was 111 MBq for both cervical and endometrial cancer. SPECT/CT imaging revealed SLNs in all patients (i.e., no non-visualizations; see Fig. 2). For cervical cancer, a total of eight SLNs were detected (median of two per patient; IQR 0.5) of which seven were also seen on lymphoscintigraphy, with bilateral drainage in 75% of the patients. For endometrial cancer, a total of 18 SLNs were detected (median of three per patient; IQR 2) of which 16 were also seen on lymphoscintigraphy, with bilateral drainage in 67% of the patients. Due to the non-standard resection locations in low-risk endometrial cancer, one SLN at the presacral region right and three SLNs at the para-aortic region were not surgically pursued, leaving 14 surgical targets.

### Intraoperative sentinel node localization

**Fig. 3:**
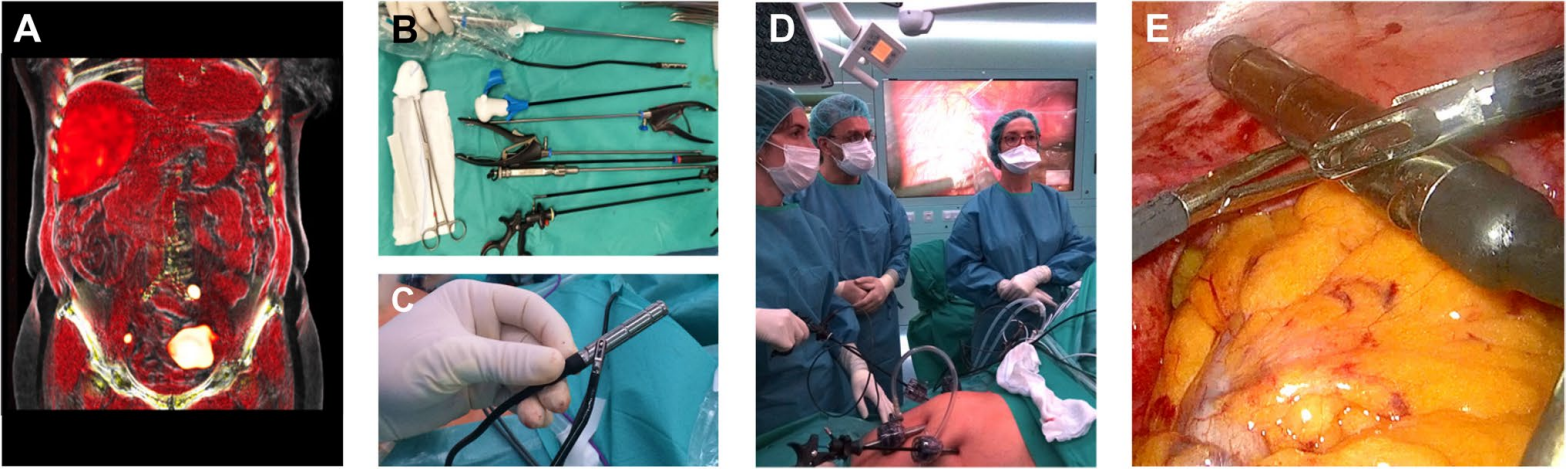
Workflow hybrid sentinel lymph node surgery with DROP-IN and fluorescence guidance for various surgical settings. **A** Surgical planning. **B**, **C** Preparation of the surgical instruments. **D** OR overview. **E** DROP-IN probe pick-up and guidance

During surgery (see Fig. 3), the DROP-IN probe was picked up by the LaproFlex instrument, with additional support from the bedside assistant either via the probe cable or via one of the additional laparoscopic instruments (Fig. 3E). The bending flexure joint of the LaproFlex, and the 45° grip design of the DROP-IN, supported a scanning range of − 90 to + 90° around the shaft of the instrument (Fig. 1B) and yielded a probe maneuverability with 6 DoF. The maximum turning circle (meaning the minimal distance needed for a complete 180° turn with the instrument positioned in its most bend state) was 15 cm. The scanning range of the traditional rigid gamma probe was fixed to a single view of 0° in front of the instrument shaft and a probe maneuverability with 4 DoF (Fig. 1A).

**Fig. 4:**
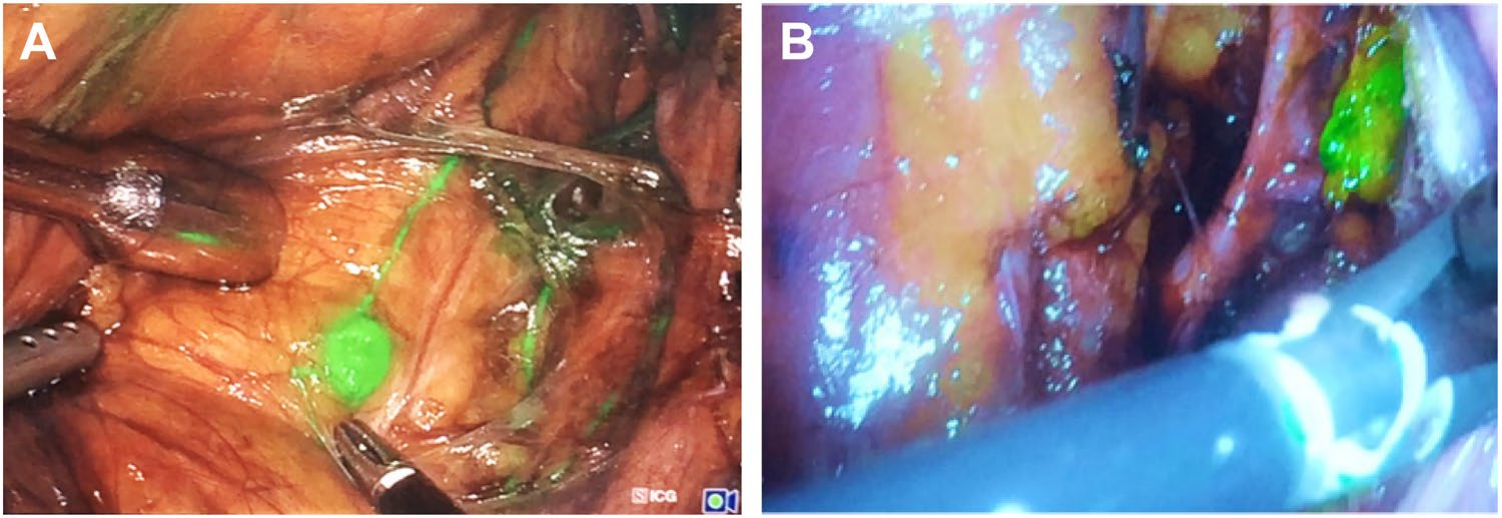
Fluorescence guidance during the hybrid sentinel node procedures in cervix and endometrial cancer. **A** Laparoscopic surgery with the Karl Storz fluorescence camera system. **B** Laparoscopic surgery with the Stryker Pinpoint fluorescence imaging system

During radioguidance in cervical cancer, SLNs were retrieved from the following anatomical locations: 10% external iliac right, 40% obturator right, 10% inter-iliac right, 10% parametrial right, and 30% obturator left. All SLN locations (100%) could be traced using either the DROP-IN + LaproFlex combination or the rigid gamma probe. However, only 70% could be detected using fluorescence (Fig. 4). During radioguidance in endometrial cancer, SLNs were retrieved from the following anatomical locations: 23% inter-iliac right, 8% iliac communis right, 8% obturator right, 15% obturator left, 8% external iliac left, 23% inter-iliac left, 8% iliac communis left, and 8% aortic bifurcation. Of all SLNs surgically pursued, 93% were successfully located with the DROP-IN + LaproFlex combination and the rigid gamma probe, failing to detect one SLN in these six patients. Unfortunately, due to logistic reasons, ICG usage was not available in this specific patient, meaning only ten SLNs were pursued with fluorescence guidance, of which 100% could be detected (Fig. 4). Overall, combining both clinical indications, this gave rise to a detection rate of 96% (DROP-IN + LaproFlex combination), 96% (rigid gamma probe), and 85% (fluorescence).

### Learning curve

The gynecologists required some training to be able to pick up and control the DROP-IN with the LaproFlex instrument, while they already had vast experience with the rigid gamma probe. Nevertheless, on average they rated their experience with the DROP-IN + LaproFlex more positive than that with the rigid gamma probe (a score of 5 versus 4, respectively). One of the reasons for this was that the increased maneuverability of the DROP-IN + LaproFlex combination made it feasible to perform gamma tracing of the SLNs without picking up background signal from the injection side. No side effects or patient complications were observed due to the DROP-IN + LaproFlex combination in vivo.

### Pathology and overall sensitivity

In general, only SLNs were retrieved that were found based on the image-guided read-out. From the ten SLNs retrieved in cervical cancer, none was found to be tumor-positive after histopathologic evaluation. From the 13 SLNs retrieved in endometrial cancer, there was one tumor-positive SLN. As such, from a clinical outcome perspective and looking at a per-patient basis, the SLN procedure itself delivered a sensitivity and negative predictive value of both 100% in this study.

## Discussion

The results from this study indicate that there is equivalence between the SLN detection achieved with a rigid laparoscopic gamma probe and the use of a DROP-IN probe with a steerable laparoscopic LaproFlex instrument. Hereby, this first-in-human evaluation (*n* = 10) was able to achieve the same accuracy as the rigid technology that the involved surgeons routinely apply for laparoscopic SLN procedures (two gynecologists > 10 years and two gynecologists > 20 years of experience). Unique to the DROP-IN + LaproFlex setup is that it provides increased flexibility and maneuverability in (non-robotic) laparoscopic surgery, a concept that is especially interesting for centers that do not have a robotic surgery program established. Moreover, we show the DROP-IN technology supports cervical and endometrial cancer SLN surgery, thereby broadening its indications.

**Fig. 5:**
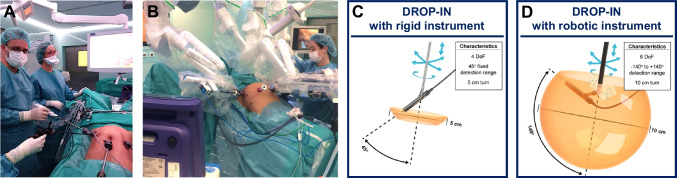
Differences in the traditional laparoscopic operating room versus robotic operating room. **A** In the traditional laparoscopic setting, trocar placement is less restricted than in the robotic setting. If the size of the trocars used permits it, this could allow for gamma probe insertion at various points of entrance in to the patient. **B** In the robotic setting, most trocars are in permanent use by the robot, limiting probe access to the assistant trocar only. **C** Schematic overview of DROP-IN probe maneuverability with rigid laparoscopic instruments. **D** Schematic overview of DROP-IN probe maneuverability with robotic instruments

The gynecologists experienced an enlarged freedom of movement when using the DROP-IN + LaproFlex combination compared to the rigid laparoscopic gamma probe. This feature helped to make it possible to better isolate the SLN signal from background signals (i.e., the injection side), yielding equivalent performance for this novel tracing approach: overall, both the DROP-IN and rigid probe achieved a 96% detection rate. While achieving equivalence to a well-established technology at an early stage of clinical implementation (*n* = 10) can be considered a positive outcome, improvement could not yet be established, a finding that seems to contradict the pronounced improved DROP-IN detection rate as reported for robotic surgery (100% versus 76%, for the DROP-IN versus rigid gamma probe respectively) [23]. Comparing the approaches shows there are two fundamental differences between non-robotic and robotic laparoscopic procedures. Firstly, in the non-robotic laparoscopic setting, probe insertion and handling are performed by the surgeon and lack of maneuverability can be overcome by inserting the probe via different trocars; however, in the current study, only the assistant trocar was large enough for probe insertion. In the robotic setting, rigid gamma probe use is often completely restricted to the assistant trocar: as the robotic platform fixes itself to the majority of the trocars (called “robot docking”), generally only one trocar is available for gamma tracing (i.e., an assistant trocar located at the side) (Fig. 5) [21], a feature that limits nodal identification on the ipsilateral side of the patient [23]. Secondly, in the robotic setting, the surgeon is no longer present at the sterile bedside, preventing autonomous tracing with the rigid probe [21], thereby making the procedure and the handling of the probe highly reliant on the communication between the surgeon and the bedside assistant. A shortcoming that is overcome with the DROP-IN probe is that this latter technology can be directly manipulated by the robotic surgeon. In addition, indicating autonomy in gamma probe use by the surgeon is a major driver for its accuracy. Prospective randomized evaluations in larger patient cohorts, and perhaps additional clinical indications, will help to further understand which features offer advantages with regard to (quantifiable) surgical outcome.

As reported by Paredes et al. and Sanches et al., the hybrid radio-and fluorescence-guidance concept enables fluorescence-based SLN confirmation in the majority of cases for both cervical and endometrial cancer [32, 33]. However, in line with previous reports [23, 24], DROP-IN radioguidance outperformed fluorescence imaging (96% versus 85%, in the current study respectively), an effect that is likely due to the in-depth SLN detection capabilities of gamma probe detection, as opposed to the superficial nature of fluorescence imaging [36].

DROP-IN usage with conventional rigid laparoscopic instruments was not evaluated in this study. As illustrated in Fig. 5C, such a setup would be possible, but would not increase the DoF available for tracing as compared to the rigid laparoscopic gamma probe (i.e., 4 DoF; Fig. 1A). Given the reported findings, one could theorize also a similar detection rate would be found with a DROP-IN + traditional instrument combination. However, looking at a different aspect of the surgical procedure, namely surgical logistics, a possible advantage of using the DROP-IN probe is also provided by the fact that it can stay in situ during the procedure, meaning it can simply be picked up whenever the surgeon needs it. A recent study indicates that kinematic assessments of the intraoperative DROP-IN probe movements can be used to objectively assess how well the probe use relates to the surgical workflow [37, 38]. Perhaps, future developments of such intraoperative kinematic assessments could eventually also be used to translate surgical preference for improved logistics and maneuverability into quantifiable surgical performance improvements as provided by the novel technologies.

There is a continuing trend towards increasingly minimal-invasive methodologies [39]. However, to realize true precision surgery, precise surgical instruments should be complemented with precise target definition by molecular detection (i.e., miniaturized detection, steerability, augmented data display) [21], innovations that are supported by the advances made in radiochemistry (i.e., novel (tumor-targeted) radiopharmaceuticals, e.g., ^99m^Tc-PSMA I&S [40], ^99m^Tc-folate [41], ^99m^Tc-FAPI-34 [42], ^99m^Tc- or ^111^In-labeled somatostatin analogs [43]). By actively pursuing both the engineering and chemical tracks, the field of radioguided surgery is rapidly changing the way surgery can be performed.

## Conclusion

We have successfully demonstrated the use of steerable DROP-IN radioguidance during laparoscopic cervical and endometrial cancer SLN procedures, thereby expanding the DROP-IN utility beyond robotic procedures. The find rate with the steerable approach was equal to that of the traditional rigid gamma probe, but came with a slightly superior surgical experience. These findings encourage further investigation and consideration on a path towards routine clinical practice and improved patient outcome.
